# Correlation between *KLOTHO* gene and mild cognitive impairment in the Uygur and Han populations of Xinjiang

**DOI:** 10.18632/oncotarget.20655

**Published:** 2017-09-06

**Authors:** Palida Abulizi, Xiao-Hui Zhou, Kabinuer Keyimu, Mei Luo, Feng-Qing Jin

**Affiliations:** ^1^ No1 Cadre Wards Medicine, The First Affiliated Hospital of Xinjiang Medical University, Urumqi, Xinjiang, 830054, China

**Keywords:** mild cognitive impairment, Klotho gene, uygur nationality, Han nationality

## Abstract

The secretive Klotho protein is an anti-aging regulatory hormone that plays a physiological role in many target organs. The present study aims to investigate the correlation between *Klotho* gene and mild cognitive impairment (MCI) in Uygur and Han populations in Xinjiang. From July 2008 to April 2014, stratified random multistage cluster sampling was used in combination with the methods of on-site questionnaire and household survey to conduct a cross-sectional MCI investigation on selected Uygur and Han subjects aged over 60 years old in Xinjiang region. 323 Uygur and Han MCI patients were randomly selected and matched with 343 subjects in the normal control group. SNaPshot technique was used to detect the polymorphisms of *Klotho* gene. A case-control associated analysis was conducted to analyze the genotype and allele frequencies of single nucleotide polymorphisms (SNPs) in the MCI group and the normal control group. The polymorphisms of rs1207568 and rs9536314/rs9527025 loci in *Klotho* gene were different among MCI populations in Xinjiang, and after group assignments based on ethnic background, the polymorphisms of rs1207568 and rs9536314/rs9527025 loci were associated with the Uygur MCI population and were not relevant to the Han MIC population. The frequencies of mutational alleles of rs9536314/rs9527025 locus in the Uygur population were significantly higher than those in the Han population. The genotype and allele frequencies of rs1207568 locus in the Uygur and Han populations were similar. The polymorphisms of rs1207568 and rs9536314/rs9527025 loci in *Klotho* gene may be associated with the Uygur MCI population in Xinjiang.

## INTRODUCTION

The *Klotho* gene is located on chromosome 13q12 and encodes a 5.2 kb transcript, with six exons [1]. There is an alternative splicing site in the third exon region producing two different transcripts. One encoding a transmembrane protein (full-length transcript, 1014 amino acids in total) with a molecular weight of about 130 kDa, which is expressed mainly in the kidney, placenta, small intestine and prostate. Another one encodes a secretive protein (truncated transcript, 550 amino acids in total) with a molecular weight of about 70 kDa, which exists in the free form in the human brain, hippocampus, placenta and kidney tissues [2]. The secretive Klotho protein is an anti-aging regulatory hormone that plays a physiological role in many target organs [3]. The *Klotho* gene is a well-recognized age-regulated gene and is significantly expressed in the distal tubule of kidney and brain choroid plexus. The extracellular domain is covered by the protease in the membrane structure and is released into the blood and cerebrospinal fluid in a secretary form, which is present as an anti-aging humoral factor. Klotho protein has many biological functions and a number of downstream targets that involve multiple signaling pathways such as the Wnt signaling pathway, the IGF-1 signaling pathway and the p53/p21 signaling pathway. Currently, *Klotho* gene has been found to have functions in energy metabolism regulation, anti-inflammation, anti-oxidation, ion transport regulation and mineral metabolism [4–6]. *Klotho* gene has 505 single nucleotide polymorphisms. The loci which receive most of research attention are located in the promoter region and exon region of the *Klotho* gene. Study found that the rs1207568 polymorphism in the promoter region of the *Klotho* gene was associated with diabetes [7], and rs9536314 and rs9527025 polymorphisms in the exon region were associated with atherosclerotic vasculopathy and coronary artery disease [8, 9] as well as osteoporosis, kidney disease and cancer. Mild cognitive impairment (MCI) is associated with many metabolic disease, such as diabetes, obesity, and cardiovascular disease. However, the relation between *Klotho* genetic polymorphisms and MCI remains unclear. Therefore, based on the preliminary epidemiological investigation of MCI, we investigated the relationship between *Klotho* gene polymorphisms and the occurrence of MCI in Uygur and Han ethnic in Xinjiang using a case-control study.

## RESULTS

### Comparison of general biochemical indicators in the two groups

Based on the preliminary epidemiological survey, a total of 323 subjects were screened out for the case group in this study, which contained 162 males and 161 females. The mean age was 75.12 ± 6.88 years, and there were 166 cases of Uygur ethnic and 157 cases of Han ethnic. There were 343 subjects in the control group, with 166 cases of women and 177 cases of men. The average age was 74.65 ± 6.17 years, and there were 172 cases of Uygur ethnic and 171 cases of Han ethnic (see Table 1). There were differences between the MCI group and the control group with respect to SBP, HC size, blood lipid levels and *Klotho* protein levels (*P* < 0.05).

**Table 1:** The comparison of general characteristics between the MCI group and the Control group

Indicators	MCI group (n=323)	Control group (n=343)	*t (x*^*2*^*)*	*P values*
Age (years)	75.12±6.88	74.65±6.17	0.937	0.345
Gender (M/F)	162/161	166/177	0.342	0.764
Alcohol (n, %)	60 (18.6)	66 (19.2)	0.331	0.860
Ethnic (Han/Uigur)	166/157	172/171	0.122	0.897
BMI (kg/m^2^)	24.39±3.89	24.11±3.70	0.946	0.345
WC (cm)	87.54±11.05	87.71±10.75	-0.085	0.933
HC (cm)	54.74±2.79	55.20±1.97	-2.445	0.015
SBP (mmHg)	137.97±20.15	132.95±19.91	3.234	0.001
DBP (mmHg)	78.59±11.46	78.47±11.23	0.138	0.890
BG (mmol/l)	6.10±2.47	6.32±2.74	-0.561	0.575
TG (mmol/l)	2.38±1.57	2.11±1.59	1.966	0.050
TC (mmol/l)	4.85±1.05	4.68±1.09	2.475	0.014
HDL-C (mmol/l)	1.21±0.33	1.37±0.54	-4.658	0.000
LDL-C(mmol/l)	2.97±0.79	2.66±0.75	5.283	0.000
*Klotho* (umol/l)	2.50±1.39	3.07±1.36	-5.405	0.000

Table 1 indicated that there are significant difference between the two groups in Klotho, LDL-C, HDL-C, TC, SBP and HC. (BMI: body mass index; WC: waist circumference; HC: Hip circumference: SBP: Systolic blood pressure; DBP: Diastolic blood pressure; BG: Blood glucose; TG: triglyceride; TC: Total cholesterol; HDL-C: High density lipoprotein cholesterol; LDL-C: Low density lipoprotein cholesterol).

### Hardy-Weinberg equilibrium (HWE) test results of the *Klotho* gene

Table 2 shows that in both Uygur and Han populations, the genotype frequencies of rs9536314 and rs9527025 loci were consistent with HWE. In the Uygur population, the distribution of allele frequency at rs9536314/rs9527025 and rs1207568 loci was in line with the law of HWE (P>0.05). Further query in the HAPMAP database revealed that the genotype frequency of the above-mentioned gene loci in the normal Han population of Beijing was 0, therefore, it was determined that the distribution pattern was consistent with the mutation frequency in the Han ethnic. The distribution of genotype frequency at rs1207568 locus was consistent with the law of genetic balance and was representative of the population (P>0.05).

**Table 2:** Hardy-Weinberg test of the genotype distribution of *Klotho* genotypes

Loci	Groups	Number of subjects (n)		SNP			*P* value
				T/T	T/G	G/G	
rs9536314/	Uygur population						
rs9527025	MCI group	166	Actual	115	43	8	
			Expected	112	49	5	0.143
	Control group	172	Actual	137	31	4	
			Expected	135	35	2	0.175
	Han population						
	MCI group	157	Actual	157	0	0	
			Expected	157	0	0	
	Control group	171	Actual	167	4	0	
			Expected	167	4	0	0.877
rs1207568	Uygur population			SNP			
				G/G	G/A	A/A	
	MCI group	166	Actual	97	62	7	
			Expected	99	59	8	0.455
	Control group	172	Actual	120	49	3	
			Expected	121	49	4	0.4278
	Han population						
	MCI group	157	Actual	108	43	6	
			Expected	107	45	5	0.5134
	Control group	171	Actual	126	43	2	
			Expected	127	41	3	0.4277

Table 2 showed these two loci were in line with Hardy-Weinberg equilibrium (all P>0.05).

### The distribution differences of genotype and allele frequencies of Klotho gene in the MCI group and the control group

Table 3 shows the frequency distribution of three loci of the *Klotho* gene in the MCI group and the control group. For rs9536314/rs9527025 locus, the distribution frequencies of TT genotype, TG genotype and GG genotype were 223 (69.04%), 86 (26.63%) and 14 (4.33%), respectively. The distribution frequencies of these three genotypes in the control group were 263 (76.68 %), 74 (21.57%) and 9 (1.75%), respectively. The distribution frequencies of these three genotypes were significantly different in the MCI group and the control group (*P* = 0.033). The distribution frequencies of the dominant model (TT vs TG + GG) in the MCI group and the control group were significantly different (*P* = 0.027). The frequency of G allele was 114 (17.65%) in the MCI group and 86 (12.52%) in the control group, and the difference was statistically significant (*P* = 0.009). The distribution frequencies of GG, GA and AA genotypes of rs1207568 locus in the MCI group were 205 (63.47%), 106 (32.82%) and 12 (3.72%), respectively. The distribution frequencies of these three genotype in the control group were 246 (71.72%), 92 (26.82%) and 5 (1.46%), respectively. The distribution frequencies of these three genotypes in the MCI group and the control group showed significant difference (*P* = 0.030), and the distribution frequencies of the dominant model (GG vs GA + AA) in the MCI group and the control group were statistically different (*P* = 0.023). The frequencies of A allele were 130 (20.12%) in the MCI group and 102 (14.87%) in the control group, respectively, and the difference was statistically significant (*P* = 0.011).

**Table 3:** Genotyping and allele distributions of *Klotho* in MCI patients and control subjects (n, %)

		MCI group (n=323)	Control group (n=343)	χ^2^ value	*P* value
rs9536314/	rs9527025				
Genotype	T T	223 (69.04)	263 (76.68)		
	T G	86 (26.63)	74 (21.57)		
	G G	14 (4.33)	6 (1.75)	6.798	0.033
Dominant model	T T	223 (69.04)	263 (76.68)		
	TG+GG	100 (30.96)	80 (23.32)	4.918	0.027
Recessive model	G G	14 (4.33)	6 (1.75)		
	TT+TG	309 (95.67)	337 (98.25)	3.816	0.051
Additive model	T G	86 (26.63)	74 (21.57)		
	TT+GG	237 (73.37)	269 (78.43)	2.325	0.127
Allele	T	532 (82.35)	600 (87.46)		
	G	114 (17.65)	86 (12.52)	6.81	0.009
rs1207568					
Genotype	G G	205 (63.47)	246 (71.72)		
	G A	106 (32.82)	92 (26.82)		
	A A	12 (3.72)	5 (1.46)	7.005	0.030
Dominant model	G G	205 (63.47)	246 (71.72)		
	GA+AA	118 (36.53)	97 (28.28)	5.183	0.023
Recessive model	A A	12 (3.72)	5 (1.46)		
	GA+GG	311 (96.28)	338 (98.54)	3.408	0.065
Additive model	G A	106 (32.82)	92 (26.82)		
	AA+GG	217 (67.18)	251 (73.18)	2.862	0.091
Allele	G	516 (79.88)	584 (85.13)		
	A	130 (20.12)	102 (14.87)	6.388	0.011

Table 3 showed that there are significant difference between the two groups in genotype and allele distribution.

### The differences in the distribution of genotype and allele frequencies of *Klotho* gene in both Uygur and Han ethnic

It can be seen from Table 4 that the frequencies of TT, TG and GG genotypes at rs3536314/rs9527025 locus of the *Klotho* gene in the Uygur MCI group were 115 (69.28%), 43 (25.90%) and 8 (4.82%). The distribution frequencies of these three genotypes in the control group were 137 (79.65%), 31 (18.02%) and 4 (2.33%), respectively. There was no significant difference in the distribution frequencies of these three genotypes between the MCI group and the control group (*P* = 0.078), but the distribution frequencies of the dominant model (TT vs TG + GG) in the MCI group and the control group were statistically different (*P* = 0.029). The frequencies of the G allele were 59 (17.78%) in the MCI group and 39 (11.34%) in the control group, respectively, and the difference was statistically significant (*P* = 0.018). The distribution frequencies of GG, GA and AA genotypes at rs1207568 locus were 97 (58.43%), 63 (41.57%) and 6 (3.62%), respectively, in the MCI group. The distribution frequencies of these alleles in the control group were 120 (69.77%), 52 (30.23%) and 3 (1.74%), respectively. There was no significant difference in the distribution frequencies of these alleles between the MCI group and the control group (P = 0.079), but the distribution frequencies of the dominant model (GG vs GA + AA) in the MCI group and the control group were statistically different (*P* = 0.030). The frequencies of A allele in the MCI group and the control group were 75 (22.59%) and 55 (15.99%), respectively, and the difference was statistically significant (*P* = 0.030). These results indicated that the distribution frequencies of rs1207568 wild type and A allele were different between the MCI group and the control group in the Uygur population.

**Table 4:** Genotyping and allele distributions of *Klotho* in the Uygur population (n, %)

Loci	Genotype	MCI group	Control group	χ^2^ value	*P* value
rs9536314/	rs9527025				
Genotype	T T	115 (69.28)	137 (79.65)		
	T G	43 (25.90)	31 (18.02)		
	G G	8 (4.82)	4 (2.33)	5.095	0.078
Dominant mode	T T	115 (69.28)	137 (79.65)		
	TG+GG	51 (30.72)	35 (20.35)	4.792	0.029
Recessive model	G G	8 (4.82)	4 (2.33)		
	TT+TG	158 (95.18)	168 (97.67)	1.534	0.216
Additive model	T G	43 (25.90)	31 (18.02)		
	TT+GG	123 (74.10)	141 (81.98)	3.068	0.080
Allele	T	273 (82.22)	305 (88.66)		
	G	59 (17.78)	39 (11.34)	5.642	0.018
rs1207568					
Genotype	G G	97 (58.43)	120 (69.77)		
	G A	63 (37.95)	49 (28.49)		
	A A	6 (3.62)	3 (1.74)	5.083	0.079
Dominant mode	G G	97 (58.43)	120 (69.77)		
	GA+AA	69 (41.57)	52 (30.23)	4.721	0.030
Recessive model	A A	6 (3.62)	3 (1.74)		
	GA+GG	160 (96.38)	169 (98.26)	/	0.330^*^
Additive model	G A	63 (37.95)	49 (28.49)		
	AA+GG	103 (62.05)	123 (71.51)	3.414	0.065
Allele	G	257 (77.41)	289 (84.01)		
	A	75 (22.59)	55 (15.99)	4.741	0.030

Table 4 showed that there are significant difference between the two groups in genotype and allele distribution. (* after an accurate Fisher validation;/indicates no data).

Table 5 shows that the distribution frequencies of TT, TG and GG genotypes at rs9536314/rs9527025 locus of the *Klotho* gene in the Han population were 157 (100%), 0 (0.00%) and 0 (0.00%) in MCI group, respectively. The distribution frequencies of the three genotypes in the control group were 167 (97.66%), 4 (2.34%) and 0 (0.00%), respectively. The distribution frequencies of the three genotypes were not statistically different between the MCI group and the control group (P = 0.245), and there was no significant difference in the frequencies of the three genetic models between the MCI group and the control group (*P* > 0.05). The frequencies of G allele in the MCI group and the control group were 0 (0.00%) and 4 (1.2%), respectively, and there was no significant difference between the two groups (*P* = 0.125). The distribution frequencies of GG, GA and AA genotypes at rs1207568 locus in the MCI group were 108 (68.79%), 43 (27.39%) and 6 (3.82%), respectively. The distribution frequencies of these alleles in the control group were 126 (73.68%), 43 (25.15%) and 2 (1.17%), respectively. There was no significant difference in the distribution frequencies of these alleles between the MCI group and the control group (*P* = 0.247), and the distribution frequencies of the three genetic models in the MCI group and the control group were not statistically different (*P* > 0.05). The frequencies of A allele in the MCI group and the control group were 55 (17.52%) and 47 (13.74%), respectively, and there was no statistically significant difference between the two groups (*P* = 0.183), indicating that the distribution frequencies of rs1207568 locus in the MCI group and the control group were similar within the Han population.

**Table 5:** Genotyping and allele distributions of *Klotho* in the Han population. (n, %)

		MCI group	Control group	χ^2^ value	*P* value
rs9536314/	rs9527025				
Genotype	T T	157 (100.00)	167 (97.66)		
	T G	0 (0.00)	4 (2.34)		
	G G	0 (0.00)	0 (0.00)	/	0.124^*^
Dominant model	T T	157 (100.00)	167 (97.66)		
	TG+GG	0 (0.00)	4 (2.34)	/	0.124^*^
Recessive model	G G	0 (0.00)	0 (0.00)		
	TT+TG	157 (100.00)	171 (97.66)	/	/
Additive model	T G	0 (0.00)	4 (2.34)		
	TT+GG	157 (100.00)	167 (97.66)	/	0.124^*^
Allele	T	314 (100.00)	338 (98.8)		
	G	0 (0)	4 (1.2)	/	0.125^*^
rs1207568					
Genotype	G G	108 (68.79)	126 (73.68)		
	G A	43 (27.39)	43 (25.15)		
	A A	6 (3.82)	2 (1.17)	2.792	0.247
Dominant model	G G	108 (68.79)	126 (73.68)		
	GA+AA	49 (31.21)	45 (26.32)	0.959	0.327
Recessive model	A A	6 (3.82)	2 (1.17)		
	GA+GG	151 (96.18)	169 (98.83)	/	0.159^*^
Additive model	G A	43 (27.39)	43 (25.15)		
	AA+GG	114 (72.61)	128 (74.85)	0.213	0.645
Allele	G	259 (82.48)	295 (86.26)		
	A	55 (17.52)	47 (13.74)	1.775	0.183

Table 5 showed that there are significant difference between the two groups in genotype and allele distribution. (*after an accurate Fisher validation;/indicates no data.)

### Difference in genotype and allele distribution frequencies of the Klotho gene between the Han and Uygur populations

From Table 6, the genotype and allele frequencies of the *Klotho* gene in the two ethnic groups were compared. The distribution frequencies of TT, TG and GG genotypes at rs9536314/rs9527025 locus of the *Klotho* gene in the MCI group of the Uygur population were 115 (69.28%), 43 (25.90%) and 8 (4.82%), respectively, and the distribution frequencies of these three genotypes in the MCI group of the Han population were 157 (100.00%), 0 (0.00%) and 0 (0.00%), respectively. There was significant difference in the distribution frequencies of these three genotypes between the Uygur and Han ethnic (*P* < 0.000). In the control group, the frequencies of TG and GG genotypes were significantly higher in the Uygur population than those in the Han population, and the frequency of the G allele in the Uygur population was significantly higher than that in the Han population (all *P* < 0.000). These results indicated that there was significant difference in the frequencies of rs9536314/rs9527025 locus in the *Klotho* gene between the Uygur and Han ethnic.

**Table 6:** Genotyping and allele distributions of Klotho between the Han and Uygur population (n, %)

Locus	Genotype	MCI group				Control group			
		Uygur population	Han population	χ2 value	P value	Uygur population	Han population	χ2 value	P value
rs9536314/9527025									
Genotype	TT	115 (69.28)	157 (100.00)			137 (79.65)	167 (97.7)		
	TG	43 (25.90)	0 (0.00)			31 (18.02)	4 (2.33)		
	GG	8 (4.82)	0 (0.00)	57.279	0.000*	4 (2.3)	0 (0.00)	27.786	0.000*
Allele	T	273 (82.23)	314 (100.00)			305 (88.66)	338 (98.83)		
	G	59 (17.77)	0 (0.000)	61.41	0.000*	39 (11.34)	4 (1.17)	30.176	0.000*
rs1207568									
Genotype	GG	97 (58.43)	108 (68.79)			120 (69.77)	126 (73.68)		
	GA	63 (37.95)	43 (27.39)			49 (28.48)	43 (25.15)		
	AA	6 (3.62)	6 (3.82)	4.116	0.128	3 (1.75)	2 (1.17)	0.735	0.693
Allele	G	257 (77.41)	259 (82.48)			289 (84.01)	295 (86.26)		
	A	75 (22.59)	55 (17.52)	2.585	0.108	55 (15.99)	47 (13.74)	0.683	0.409

(* indicates *P* < 0.000).

By comparing the rs1207568 locus of the *Klotho* gene in the MCI group of different ethnic, it was found that the distribution frequencies of GG, GA and AA genotypes in the Uygur population were 97 (58.43%), 63 (37.95%) and 6 (3.62%), respectively. The distribution frequencies of these genotypes in the Han population were 108 (68.79%), 43 (27.39%) and 6 (3.82%), respectively. There was no significant difference in the frequencies of the three genotypes among the Uygur and Han populations (*P* = 0.128). In the MCI group, the frequency of the A allele was 75 (22.59%) and 55 (17.52%) in the Uygur and Han populations, respectively, and the difference was not statistically significant (*P* = 0.108). In the control group, the frequencies of different genotypes and the alleles in the Uygur and Han populations were similar and the difference was not statistically significant (*P* > 0.05), indicating that the distribution frequencies of rs1207568 locus in Uygur and Han populations were similar.

### The association between the genotypes and genetic models of the *Klotho* gene polymorphism and MCI

Table 7 shows the correlation between MCI and the two loci of the *Klotho* gene (rs9536314/rs9527025 and rs1207568), and the correlation between MCI and the polymorphisms at the two loci of the *Klotho* gene was investigated using univariate and multivariate unconditional Logistic regression analyses. The results from the univariate analysis showed that rs9536314/rs9527025 and rs1207568 loci were not associated with MCI, and the multivariate analysis also showed that there was no correlation between rs9536314/rs9527025 and rs1207568 loci and MCI when the results were adjusted for age, gender, ethnicity and alcohol consumption (*P* > 0.05).

**Table 7:** The Relationship of *Klotho* Genotype, Genetic Models, and MCI

Polymorphism locus/genetic model	Genotype	*OR* (95%CI)	After adjustment *OR* (95%CI)^#^
rs9536314/rs9527025			
Genotype	T T	1	1
	T G	1.309 (0.829-2.068)	1.293 (0.791-2.112)
	G G	2.775 (0.534-14.424)	2.904 (0.552-15.260)
Dominant model	T T	1	1
	TG+GG	1.381 (0.887-2.149)	1.371 (0.851-2.208)
Recessive model	G G	1.637 (0.718-3.730)	1.654 (0.723-3.783)
	TT+TG	1	1
Additive model	T G	0.772 (0.489-1.219)	0.792 (0.485-1.292)
	TT+GG	1	1
rs1207568			
Genotype	G G	1	1
	G A	1.131 (0.767-1.667)	1.124 (0.721-1.750)
	A A	1.056 (0.741-1.503)	1.057 (0.601-1.858)
Dominant model	G G	1	1
	GA+AA	1.086 (0.793-1.487)	1.110 (0.719-1.713)
Recessive model	A A	1.001 (0.855-1.173)	0.981 (0.787-1.224)
	GA+GG	1	1
Additive model	G A	0.907 (0.641-1.283)	0.914 (0.645-1.294)
	AA+GG	1	1

(# adjusted for age, gender, ethnic and SBP).

## DISCUSSION

The minor allele frequency of rs9536314 locus is 17% in European population, but is 0% in Beijing population of China [10]. Our results indicated that the overall minor allele frequency of the loci in the Uygur population is 17.78%, which is similar to that in Native American and Italian populations (16%) [10]. The minor allele frequency in the Han population is 1.20%, which is slightly higher than that of the Beijing population. The minor allele frequency of rs9527025 locus is 16% in European population, but 0.00% in the Beijing population and in Japanese population [11]. However, the minor allele frequency in the Uygur population is 17.78%, which is similar to that in European population.

The results of this study showed that the mutation frequencies of rs9536314/rs9527025 and rs1207568 loci in the MCI group and the control group were different, and the difference was mainly in the Uygur population. In the Han population, the mutant gene frequencies in the MCI group and the control group were similar. The mutation frequencies of rs9536314/rs9527025 and rs1207568 alleles in the MCI group of the Uygur population were all significantly higher than those in the control group of the Uygur population (*P* < 0.05), and there was significant difference between the Uygur and Han ethnic.

The *Klotho* gene is an anti-aging gene and was discovered in 1997 by Kuro-o et al. [12]. *Klotho* gene-deficient mice developed symptoms similar to those which occurred during human aging, such as atherosclerosis, ectopic calcification, emphysema, decreased motility, gonadal dysplasia, skin atrophy, hypoglycemia and severe hyperphosphatemia, whereas in mice overexpressing the *Klotho* gene, the symptoms of aging can be delayed [13]. MCI is thought to be a form of pathological aging, and the study of the association between the *Klotho* gene and cognitive impairment and AD will no doubt bring new opportunities and hopes by adding a new direction in anti-aging research.

There are a number of epidemiological evidences from large scale studies showing that the *Klotho* gene polymorphism is associated with cognitive functions. By studying a Danish population of 1480 elderly subjects (92 to 100 years old), Mengel-From et al. investigated the relationship between 19 SNPs in the *Klotho* gene and cognitive functions [14]. It was found that the rs2283368 and rs9526984 SNPs in the *Klotho* gene were associated with more than 7 years of cognitive decline. A British study on the relationship between *Klotho* genotypes and cognitive ability showed that [15] after a joint analysis of Lothian and Aberdeen cohorts, an interaction between *Klotho* and gender was found. When the cognitive abilities at age 11 were adjusted for, the nonverbal inference scores of 79-year-old females carrying a V/V genotype were lower, suggesting that the *Klotho* gene polymorphism was associated with the ability of reasoning. Specifically, poor non-verbal reasoning ability is seen in elderly females carrying the V/V genotype, suggesting that human *Klotho*-VS polymorphism is associated with cognitive decline and shortened life span. In 2006, a polymorphism investigation was carried out for the G395A locus of the *Klotho* gene in a Japanese population aged 40-79 years old, and it was found that in people aged over 60 years, the association between G395A of *Klotho* gene and cognitive function was statistically significant [16].

In this study, the allele frequencies of rs3536314/rs1207568 locus in the *Klotho* gene were significantly different between the MCI group and the control group (*P* < 0.05). The mutation frequencies of the alleles at rs9527025 locus were statistically significant (*P* < 0.05), suggesting that the risk of MCI in Xinjiang population is related to the mutations at rs9536314 locus (F352V), rs9527025 locus (C370S) and rs1207568 locus (G395A). However, after sub-grouping the subjects based on their ethnical backgrounds, the mutation frequencies in the Uygur population were much higher than those in the Han population, which indicated that the genetic background difference was an issue to be considered in the MCI population. Further multivariate regression analysis showed that the polymorphisms at three loci of the *Klotho* gene were not an independent risk factor for the MCI population in Xinjiang.

Some Chinese studies have shown that [17] the *Klotho* protein exerts a certain degree of protection against the occurrence and development of aging diseases in the central nervous system. In the first part of this study, it was found that the expression of *Klotho* protein in MCI patients was lower than that in normal controls, and the *Klotho* protein was a protective factor against MCI. This result is in agreement with the results on the relation between *Klotho* gene polymorphism and cognitive impairment obtained by Chinese and foreign researchers. However, it was also found that subjects carrying a KL-VS heterozygous genotype had a better cognitive ability and a tendency of longevity. Arking et al. [18] also found that in a Jewish population aged above 80 years in Central Europe, the subjects carrying a KL-VS heterozygous genotype showed the advantage of longevity. In a study conducted by Yokoyama et al. [19], the relationship between the KL-VS polymorphism and the human cortical volume was studied and it was found that the KL-VS heterozygous carriers had a larger sized right posterolateral prefrontal cortical volume, which contributed to an increase in executive capacity. Therefore, the *Klotho* gene was associated with a greater brain volume and a better cognitive function. The mutations in *Klotho* gene functions are related to human aging, indicating that *Klotho* gene mutations affect human life.

Animal experiments have further revealed the mechanisms of anti-aging functions of the *Klotho* gene. Studies on transgenic mice carrying a human amyloid precursor protein (hAPP) and *Klotho* genes [20] have shown that an increase in *Klotho* expression could reduce premature death of hAPP mice and improve the functions of brain network. Increasing the level of *Klotho* could prevent the depletion of NMDA receptor subunits in the hippocampus and increase the spatial learning and memory ability of hAPP mice. Therefore, increasing or activating the level of wild-type *Klotho* may increase the synaptic and cognitive functions, and may be beneficial in the treatment of AD or other types of cognitive impairments. In addition to suppress insulin signaling and cause an increase in its function against oxidant stress, *Klotho* also facilitates oligodendrocyte maturation and the formation of medullary sheath [21]. *Klotho* secreted by neurons and choroid plexus acts as a hormone-like factor in the development of the nervous system and promotes the formation of medullary sheath. *Klotho* maintains and supports the functional homeostasis of oligodendrocytes and oligodendrocyte progenitors. Down-regulation of *Klotho* in senile white matters may impair medullary sheath and cause age-related cognitive decline. Increasing the level of *Klotho* protects the integrity of phospholipids in the myelin sheath and prevents myelin degeneration in elderly brains. Thus, as a new member of the protein family, *Klotho* is essential in the connection between neurons and oligodendrocytes. *Klotho* enhancers may be a new therapeutic approach in the treatment of neurodegenerative diseases [22].

In summary, the results from this study found that there was ethnic difference in mutation frequencies of rs9536314, rs9527025 and rs1207568 loci. Due to genetic background difference, the mutation frequencies in the Han population were very low, and the multivariate regression analysis showed that the mutations at rs1207568 and rs9536314/rs9527025 loci were not related to the occurrence of MCI in Xinjiang. However, the specific mechanisms need to be further studied.

## MATERIALS AND METHODS

### Subjects

All the patients and controls selected for the present study were from an epidemiological survey. This survey adopted a stratified random multistage cluster sampling method to conduct a cross-sectional survey of MCI population in Xinjiang. All subjects were Uygur and Han ethnic who were aged over 60 years old and were from Moyu county and 6 surrounding townships and communities in Hetian region as well as from Xin Nongda, He Gang, Tielu Ju and Si Jian communities in the city of Urumqi. The survey was conducted on data between July 2008 and April 2014 and included a total of 3346 Uygur people and 1780 Han people. After strict screening based on epidemiological investigation and with reference to the diagnostic criteria of MCI listed in the fourth revised edition of Diagnostic and Statistical Manual of Mental Disorders (DSM-IV) published by American Psychiatric Association, a total of 157 cases of Han ethnic and 166 cases of Uygur ethnic were obtained. Briefly, all the patients must meet the following criteria: (1) subjective feeling of memory loss; (2) objective evidences of MCI by objective examination, such as MMSE score 18 ∼ 21 (illiterate), 21 ∼ 24 (primary school education level), 25 ∼ 27 (secondary education), and the GDS score was 2 to 3; (3) social life ability functional decline; (4) HIS ≤4, the exclusion of certain reasons caused by the decline of cognitive function; (5) the course of more than 3 months; (6) do not meet the diagnostic criteria for dementia (CDR =0.5 scale). Each case is in accordance with the above 6 diagnostic criteria, and by the Xinjiang Medical University Geriatrics Research Group experts according to the medical history, physical examination, the results of a comprehensive analysis of the scale, to make a diagnosis.

We excluded the subjects with history of mental problems or congenital mental retardation, severe cardiopulmonary, liver or kidney dysfunction, severe endocrine diseases, severe infectious diseases or toxic encephalopathy diseases. The people with neurological diseases that can cause brain dysfunctions, such as stroke, Parkinson's disease or brain tumors, depression, a history of head trauma or a history of special drug usages, and with alcohol or drug dependence within the past 6 months, were also excluded from this study.

The control subjects were selected randomly from the region of epidemiological survey and matched the age, sex and ethnic of the case group. The selected subjects in the control group had following characteristics: according to MMSE score scale: illiteracy ≥ 21 points, primary school ≥ 24 points, junior high school and above ≥ 27; the CDR scale = 0; the ADL scale was less than 16 points. There were 171 cases of Uygur ethnic and 172 cases of Han ethnic in the control group.

In the MCI group and the control group, the gender difference was not statistically significant as verified by the χ^2^ test. The age difference was not statistically significant as verified by the *t* test. All subjects signed written consent. The protocol was reviewed and approved by the Medical Research Ethics Committee of Xinjiang Medical University.

### Determination of biochemical and other indicators

After being fasted for 12 h, 5 ml of venous blood was collected from each subject in the morning. The serum was separated and conventional biochemical indicators such as blood lipids and blood glucose were measured using an automatic biochemical analyzer (Beckman, USA).

During the physical examination, weight, height, and waist circumference (WC), hip circumference (HC), and blood pressure were measured in a standardized fashion [23]. BMI was calculated as weight in kilograms divided by the square of the mean height in metres (kg/m^2^).

### DNA extraction

In each sample, 5 ml of venous blood was taken and underwent anticoagulation using ethylenediaminetetraacetic acid (EDTA). The peripheral blood genomic DNA was extracted using a whole blood genomic DNA extraction kit from Tiangen.

### Multiplex PCR amplification

Primers of the *Klotho* gene (C677T, A1298C, G1968A) were designed using the Primer 3 software (http://frodo.wi.mit.edu/), and the primers were synthesized by Shanghai Biotechnology Co., Ltd. (See Table 8).

**Table 8:** Extension primer sequences

Primer name	Sequence(5'to3')	Target amplification segment (bp)
rs9536314/rs9527025F	GTCCCACTCAGGGAGGTCAGGT	342
rs9536314/rs9527025R	TCCAGGAAAGCAGTTGCCTCAG	
rs1207568F	TAGGATTTCGGCCAGTCCCTAA	243
rs1207568R	TTCGTGGACGCTCAGGTTCATT	

### PCR product purification

After the PCR amplification, 5U SAP enzyme and 2U Exonuclease I enzyme were added into 15 μl of PCR products and incubated in a 37°C water bath for 1 h, followed by 75 °C inactivation for 15 min.

### SNaPshot extension reaction

Four SNaPshot extension primers were designed, and the 3' end of the primers was located at the first base upstream of the polymorphism locus. The 5' end of the primers contained different lengths of polyC to make the product lengths between 20 and 50 bp, in order to facilitate the detection (see Table 9).

**Table 9:** Extension primer sequences for SNP

Primer name	Sequence (5'to3')
rs1207568SR	GAAAAGGCGCCGACCAACTTT
rs9536314SF	TTTTTTTTTTTTTTTTTTTTTGAATAACCTTTCATCTATTCTGCCTGAT
rs9527025SR	TTTTTTTTTTTTTTTTTTTTTTTTTTTTTTGAAAACTCAAGGTGGGTCCAAAG

### Genotyping by capillary electrophoresis detection

**Figure 1:**
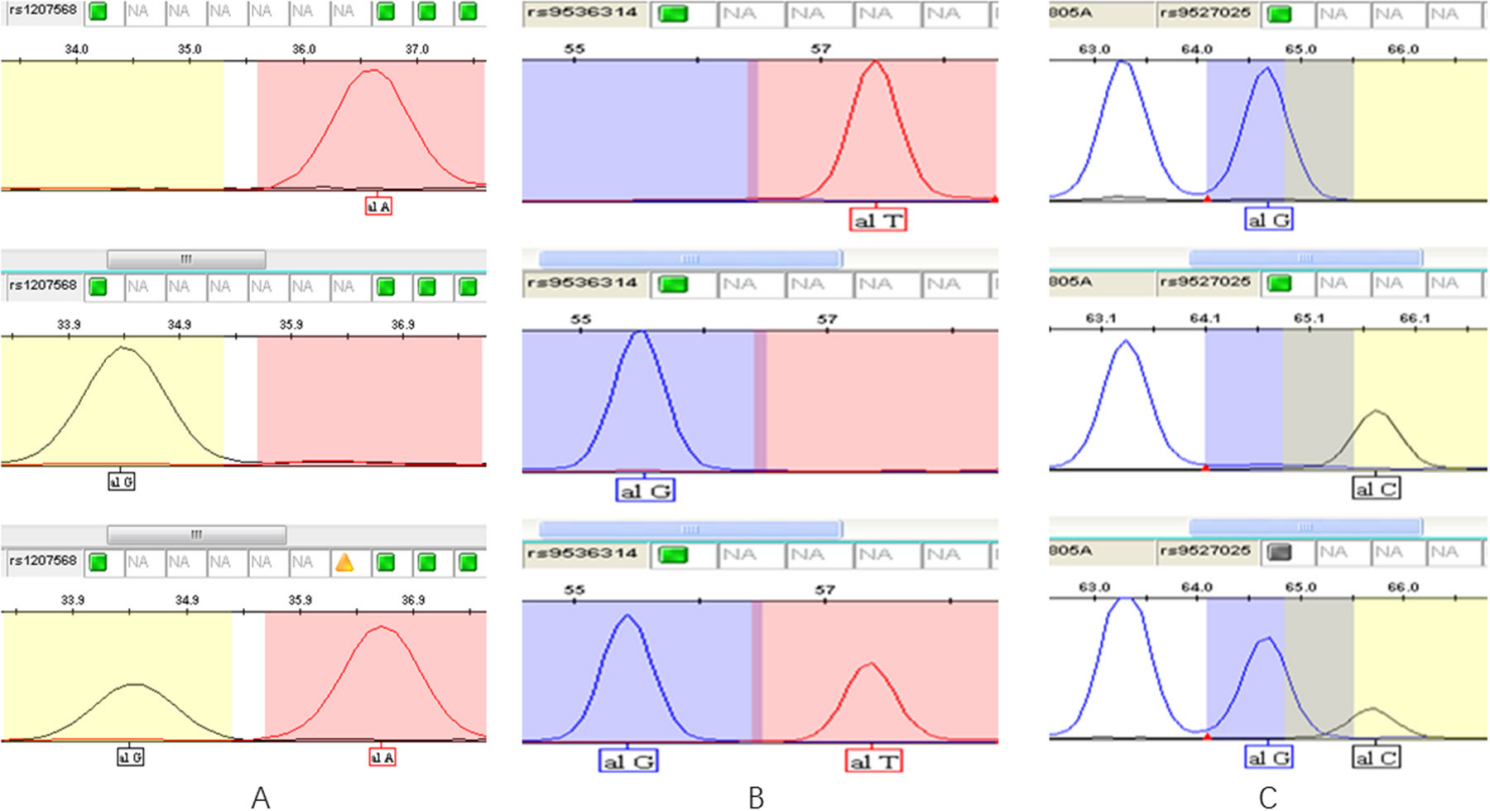
Genotyping results of three SNPs **(A)** rs1207568 (Upper: AA; Middle: GG; Lower: AG); **(B)** rs9536314 (Upper: TT; Middle: GG; Lower: TG); **(C)** rs9527025 (Upper: TT; Middle: GG; Lower: TG).

0.5 μl of the purified extension product was mixed with 0.5 μl of internal standard Liz120 (ABI) and 9 μl of formamide. After denaturation at 95°C for 5 min, the samples were put into an ABI 3130XL sequencer. The original data collected by ABI 3130XL were analyzed using the GeneMapper 4.1 (Applied Biosystems Co., Ltd., USA) software. Genotyping results are shown in Figure 1.

### Statistical analysis

All experimental data were entered a database in the SPSS17.0 software. All measurement data were presented as $\bar{x}$ ±*s*, and the comparisons of the measurement data between groups were tested using *t* test. χ^2^ test was used to detect and verify whether the genotype frequency distributions in the MCI group and the control group were consistent with the Hardy-Weinberg equilibrium. χ^2^ test was used to verify the comparison of genotype and allele frequencies between the two groups, and odds ratio (OR) and the 95% confidence intervals (CI) were calculated to estimate the relative risk of genotype mutation on the occurrence of the disease. To adjust the confounders, such as SBP, gender, ethnic, and age, a logistic regression analysis was carried out. A significance level of α = 0.05 was used for statistical analysis.
